# Could Lipoxins Represent a New Standard in Ischemic Stroke Treatment?

**DOI:** 10.3390/ijms22084207

**Published:** 2021-04-19

**Authors:** Nikola Tułowiecka, Dariusz Kotlęga, Andrzej Bohatyrewicz, Małgorzata Szczuko

**Affiliations:** 1 Department of Human Nutrition and Metabolomics, Pomeranian Medical University, Broniewskiego 24 Street, 71-460 Szczecin, Poland; ntulowiecka97@gmail.com; 2 Department of Neurology, District Hospital, 67-200 Głogów, Poland; dkotlega@uz.zgora.pl; 3 Department of Orthopaedics, Pomeranian Medical University, Żołnierska 48, 71-210 Szczecin, Poland; andrzej.bohatyrewicz@pum.edu.pl

**Keywords:** lipoxins, arachidonic acid, inflammation, cardiovascular disease, ischemic stroke

## Abstract

Introduction: Cardiovascular diseases including stroke are one of the most common causes of death. Their main cause is atherosclerosis and chronic inflammation in the body. An ischemic stroke may occur as a result of the rupture of unstable atherosclerotic plaque. Cardiovascular diseases are associated with uncontrolled inflammation. The inflammatory reaction produces chemical mediators that stimulate the resolution of inflammation. One of these mediators is lipoxins—pro-resolving mediators that are derived from the omega-6 fatty acid family, promoting inflammation relief and supporting tissue regeneration. Aim: The aim of the study was to review the available literature on the therapeutic potential of lipoxins in the context of ischemic stroke. Material and Methods: Articles published up to 31 January 2021 were included in the review. The literature was searched on the basis of PubMed and Embase in terms of the entries: ‘stroke and lipoxin’ and ‘stroke and atherosclerosis’, resulting in over 110 articles in total. Studies that were not in full-text English, letters to the editor, and conference abstracts were excluded. Results: In animal studies, the injection/administration of lipoxin A4 improved the integrity of the blood–brain barrier (BBB), decreased the volume of damage caused by ischemic stroke, and decreased brain edema. In addition, lipoxin A4 inhibited the infiltration of neutrophils and the production of cytokines and pro-inflammatory chemokines, such as interleukin (Il-1β, Il-6, Il-8) and tumor necrosis factor-α (TNF-α). The beneficial effects were also observed after introducing the administration of lipoxin A4 analog—BML-111. BML-111 significantly reduces the size of a stroke and protects the cerebral cortex, possibly by reducing the permeability of the blood–brain barrier. Moreover, more potent than lipoxin A4, it has an anti-inflammatory effect by inhibiting the production of pro-inflammatory cytokines and increasing the amount of anti-inflammatory cytokines. Conclusions: Lipoxins and their analogues may find application in reducing damage caused by stroke and improving the prognosis of patients after ischemic stroke.

## 1. Introduction

Cardiovascular diseases including myocardial infarction and stroke are the most common causes of mortality in the USA [1]. There are three types of stroke: ischemic stroke (IS), intracerebral hemorrhage (ICH), and subarachnoid hemorrhage. Ischemic strokes account for approximately 80% of all strokes. Intracranial hemorrhages, and more specifically subarachnoid hemorrhage (SAH), represent subtypes of stroke that are associated with high mortality and disability [2]. These three types of stroke are completely different pathophysiologically and clinically as disease entities. SAH in most cases results from rupture of intracranial aneurysms, while ICH results from interstitial disruption of blood vessels. ICH most often occurs due to the degeneration of intracerebral arteries, with the highly significant influence of arterial hypertension and alcohol abuse as the main risk factors. SAH and ICH differ between ischemic stroke not only in pathophysiology but also in the extent of brain damage. In SAH, blood extravasates primarily, and secondarily, there is an increase in intracranial pressure, edema of the brain, and hydrocephalus. Focal brain damage may occur in the subacute period of the disease secondary to vascular spasm. With regard to ICH, the difference between ischemic stroke is related to the occurrence of primary focal brain damage. Then, the inflammatory cascade is triggered. It is additionally activated by the irritating effect of the blood within the hematoma, erythrocyte aggregation, hemostasis and coagulation cascade, an increase in blood–brain barrier (BBB), intracranial pressure, and swelling of the brain. Neuroinflammation plays an important role in the evolution of the hemorrhagic focus [3,4].

Early brain injury has been identified as the leading cause of mortality in patients with SAH and has been identified as the primary goal of treatment [2,5,6]. In ischemic stroke, the main pathogenetic factor is an inflammation caused by atherosclerosis—a chronic, progressive inflammatory state characterized by vasculitis and the accumulation of lipids beneath the inner surface of endothelium. Atherosclerotic plaques can appear even in young people and are asymptomatic for years [1,7]. The stable structure of the atherosclerotic plaque gradually narrows the lumen of the vessel, which results in impaired blood flow [8]. The progression and intensification of the inflammatory process taking place in the vessel wall cause destabilization of the plaque. Such an abnormal process causes instability of atheroscleroitc plaque and rupture [8]. Interferon-γ (IFN-γ) plays a significant role in the destabilization of the plaque, inactivating myocytes and inhibiting their proliferation and collagen synthesis, while stimulating metalloproteinase-producing macrophages (MMPs) responsible for the degradation of connective tissue structures [8]. When the atherosclerotic plaque is ruptured, an acute ischemic episode such as a heart attack or stroke may occur [1,9]. There are some common elements in the inflammatory cascade of intracranial bleeding and ischemic stroke. However, due to the completely different pathophysiological background, clinical picture of the patient, and the treatment used, in this work, we focused on the analysis of important pathogenetic elements and the possibility of using lipoxins in patients with ischemic stroke. Analysis of the potential benefits of lipoxins in terms of ICH and SAH requires separate analysis. Neurons die very quickly during stroke due to energy deficit, ion imbalance, mitochondrial failure, and activation of intracellular lipases, proteases, and ribonuclease, which cause rapid breakdown of the cell’s structural elements and its integrity [10]. The immune response begins in the vessel and, in the case of an ischemic stroke, in ischemic brain cells. When vascular obstruction occurs, an inflammatory cascade is triggered. Platelets are activated, which bind to P-selectin molecules, causing clogging of the vessels and additionally contributing to brain damage [10]. Inflammatory mediators spread throughout the body, causing inflammation to suppress the potentially harmful, pro-inflammatory environment [10]. Uncontrolled inflammation is also associated with other cardiovascular diseases, neurodegenerative diseases, diabetes, obesity, asthma, and inflammatory diseases (including arthritis). The inflammatory reaction produces chemical mediators that stimulate the regression of the inflammatory response [11,12]. Endogenous anti-inflammatory mediators identified in recent years include resolvins, protectins, and maresins from the omega-3 fatty acid family, as well as derived from an omega-6 family and arachidonic acid (AA)—lipoxins.

When analyzing the pathophysiology and potential use of lipoxins in stroke therapy, the role of brain energy metabolism, edema, and the inflammatory cascade in the brain should also be considered.

## 2. Energetic Metabolism in Brain

The brain is an organ with an extremely high-energy requirement. It consumes about 20% of the total oxygen requirement and about 25% of total glucose consumption [13]. Therefore, the functioning of the brain is dependent on the constant receiving of energy substrates from the blood. The blood–brain barrier is made up of endothelial cells, their junctions, pericytes, basement membrane, and astrocyte end feet. BBB prevents the flow of fluids between the blood and the brain, and it allows the penetration of essential nutrients toward the brain and the elimination of metabolic products outside. An important role of the BBB is also to enable the penetration of nutrients, such as amino acids or glucose, and to prevent the penetration of blood-derived cells, such as, for example, lymphocytes, which could disturb the function of neurons [14]. Within the BBB, there are two main types of transporters for fatty acids, carbohydrates, monocarboxylic acid, amino acids, and nucleotides, as well as for organic cations and anions. These are the solute carrier (SLC) proteins and the adenosine triphosphate (ATP)-binding cassette (ABC) proteins [15]. Glucose metabolism in the brain proceeds through the synthesis of ATP by glycolysis, tricarboxylic acid cycle, and the pentose phosphate pathway. Glucose transporters (GLUTs) are part of the SLCs proteins and are especially important for proper energy metabolism. Glucose metabolism within the central nervous system takes place in neurons and in astrocytes. Glucose enters neurons through the GLUT3 transporter and to astrocytes through GLUT1. Neurons are unable to store glucose in the form of glycogen because of constitutive degradation of glycogen synthase. Astrocytes are able to convert glucose into glycogen, which is the most important source of energy for the brain. Pericytes, which are located perivascularly, participate with astrocytes in maintaining glucose metabolism [16]. The occurrence of ischemic stroke results from cerebral circulation disturbance, which leads to the disintegration of cell membranes and disorders in the functioning of the mitochondria. This is followed by the inflammatory cascade, oxidative stress, destruction of the BBB, the excytotoxic reaction, and then cell death [17]. Neurons die very quickly during stroke due to energy deficit, ion imbalance, mitochondrial failure, and activation of intracellular lipases, proteases, and ribonuclease, which cause rapid breakdown of the cell’s structural elements and its integrity [18].

## 3. Cytotoxic and Vascular Edema

Following cerebral ischemia, astrocyte swelling is one of the earliest responses. The regulation of astrocyte cell volume is crucial to avoid the negative consequences of post-stroke edema [19]. This has been precisely shown recently by Kitchen et al. and their earlier study [20,21]. Cytotoxic edema includes oncotic cell swelling caused by the movement of osmotically active molecules (mainly Na^+^, Cl^−^, and water) from the extracellular space to the intracellular space. The extracellular space of the brain is relatively small compared to the intracellular space—it accounts for about 12–19% of the brain’s volume [22]. Edema appears to be a general response of astrocytes to injury and appears quickly after various types of CNS injury, including ischemia, trauma, hypoglycemia, status epilepticus, and liver failure [23]. The second phase of endothelial dysfunction is characterized by the breakdown of the BBB with the leakage of plasma proteins into the extracellular space. This is called angioedema, and it occurs later in the stroke. Macromolecules such as albumin, IgG, and dextran, for which the blood–brain barrier is normally impermeable, begin to easily cross the endothelial barrier [22]. The presence of edema plays an important role in the prognosis of stroke patients. Brain edema occurs as a result of the destruction of the BBB and the ingress of water into the central nervous system. The aquaporin 4 (AQP4) water channel is of great importance in the process of water transport to the CNS [24]. The third phase of endothelial dysfunction is characterized by catastrophic damage to the integrity of the capillaries, during which all blood components, including red blood cells, are extravasated into the parenchyma of the brain. Hemorrhagic conversion—transformation of an ischemic focus into a hemorrhagic infarction after circulation is restored—is the main cause of early mortality in patients with acute stroke [23].

## 4. Inflammation in Ischemic Stroke

During ischemic stroke, an inflammatory reaction occurs, which is caused by insufficient supply of oxygen in its structures. The cells that penetrate the hypoxic tissue most rapidly are neutrophils. The infiltration of neutrophils occurs within the first hours after the restoration of blood flow. In the following days, monocytes and macrophages also migrate into the hypoxic tissue. The activity of inflammatory cells at the site of hypoxia is not elucidated in detail, but it is known that they lead to impairment of the nervous tissue function through the production of free radicals, cytokines, and nitric oxide [25]. Thus, the neutrophils begin the process of penetrating the inflammation focus in the first hours of the infarction, and they significantly contribute to the enlargement of the area of the cerebral infarction. Monocytes accumulate in the first 24 h, peaking at 48 h. At the very end, lymphocytes appear, approximately 24–48 h after the onset of the stroke [26]. The blood–brain barrier acts as a barrier that separates the central nervous system (CNS) from peripheral tissues. Its permeability depends on the action of endothelial cells [27]. Damage to the BBB occurs as a result of the action of matrix metalloproteinases (MMPs). Damage to the BBB changes its permeability to leukocytes that activate pro-inflammatory processes. As a consequence, swelling of the cerebral vessels and an increase in intracranial pressure occur. The pathophysiology of the edema itself is complex and depends on the type of factor causing the brain damage and the individual response of the patient [19]. Inflammation caused by an acute cerebral ischemia stimulates the development of the molecular pro-inflammatory mechanism, influencing the functioning of the CNS and the vascular system. Microglia plays an important role among CNS cells, the scope and activation effect of which depends on the state of brain damage and the duration of ischemia. The activated microglia produces neurodegenerative and pro-inflammatory factors. During cerebral ischemia, the expression of endothelial adhesion molecules for leukocytes is increased, and the components of selective transport of molecules are activated [25,26,27]. However, despite promising results in experimental studies, inflammation-modulating treatments have not yet been translated successfully into the clinical setting.

## 5. Treatment of Ischemic Stroke

### 5.1. Present Treatment of Stroke

The treatment of ischemic stroke is based on the introduction of causal treatment (i.e., thrombolytic and endovascular treatment). In the specific treatment of ischemic stroke, intravenous thrombolysis with recombinant tissue plasminogen activator (rt-PA, alteplase) is used, which is recommended in the treatment of acute ischemic stroke up to 4.5 h after the onset of symptoms, and preferably as soon as possible, because the greatest effectiveness of thrombolytic treatment is obtained in the first hour after the onset of symptoms [28].

Maintaining elevated blood pressure in order to maintain normal cerebral blood flow is essential for the effective treatment of ischemic stroke. In the acute phase of ischemic stroke, it is recommended to lower blood pressure only when systolic blood pressure is >220 or diastolic >110 mmHg or thrombolytic treatment is introduced [29]. In the case of circulatory failure and hypotension, drugs from the group of catecholamines such as dobutamine are used (it causes an increase in cardiac output but does not significantly change the heart rate and blood pressure), dopamine, which is essential in people with renal failure or hypotension, as well as norepinephrine for septic shock [28,30].

### 5.2. Inflammation and Classic Anti-Inflammatory Treatment

Stroke-induced inflammatory processes, which include mechanisms of innate and adaptive immunity, are a response to tissue damage due to the absence of blood supply but have also been proposed as key contributors to all the stages of the ischemic stroke pathophysiology [31].

There are differences in the relationship between IS and current NSAIDs. The risk of IS appears to be higher in patients with a prior history of IS or transient ischemic attack (TIA), in younger or male patients. Concomitant use of aspirin, anticoagulants, and platelet aggregation inhibitors appears to mitigate this risk. Both NSAIDs and coxibs might increase the risk of IS, suggesting that pharmacological properties other than COX-2 selectivity are important [32].

Antiplatelet drugs, including the acetylsalicylic acid (ASA), are used in the secondary prevention after stroke. If the patient was previously treated with rt-PA, then the antiplatelet drug may be administered 24 h after the end of thrombolytic therapy [33]. ASA has a wide range of use in the prevention of patients with known cardiovascular disease. The preventive effect is primarily related to the ability to inactivate two isoenzymes—cyclooxygenase 1 (COX-1) and cyclooxygenase 2 (COX-2), which leads to inhibition of thromboxane 2 (TXA2) production. COX 1 has a protective effect on the gastric mucosa and also has a protective effect on blood vessels [34,35].

COX 2 is involved in the inflammatory response and the formation of prostaglandins, thromboxanes and prostacyclin. Prostacyclins together with leukotrienes and thromboxanes are involved in platelet aggregation, fibrinolysis, the process of contraction, and the relaxation of blood vessels, as well as the inflammatory response. The influence of ASA on all types of COX is irreversible [36]. Consequently, the inhibition of COX is sustained throughout the platelet life of 10 days. Acetylsalicylic acid is absorbed in the stomach and upper small intestine, but it can also occur in the mouth when the tablet is chewed rather than swallowed, allowing blood levels to be obtained in the blood to inhibit platelets in a shorter time. In the case of coated tablets, the highest concentrations of ASA are obtained after about 3–4 h after ingestion of the drug, while uncoated tablets allow this to be achieved after about 30–40 min. ASA is largely excreted via the kidneys [37].

The potential use of anti-inflammatory treatments gives rise to the controversy about the adverse effects of use (including also the tranexamic acid); therefore, safer treatments such as lipoxin can present themselves as future therapeutic options.

### 5.3. Potential Role of Aquaporins in Stroke Treatment

Of great hope are the proteins aquaporin (AQP), which create channels for the flow of water and therefore play an important role in the development of edema, especially the AQP4, which is abundantly expressed in astrocytes. AQP also facilitate physiological processes by mediating the diffusion of small, neutral solutes [38]. AQPs also play a role in mediating the osmotic movement of water in regulating cell volume, which allows cells to change their size. Research shows that this role may extend beyond mere passive water pores and may involve signal transduction through them [39]. Moreover, it has been shown that AQP4 expression coincides with areas of glial-specific edema and distribution [40]. Studies suggest that AQP4 plays a role in the resolution of CNS edema, with the flow of water through AQP4 accelerating the development of cytotoxic edema in the early stage after trauma and later eliminating angioedema [21]. Kitchen et al. show an increased abundance of AQP4 at the cell membrane in response to hypoxia-induced cellular edema in a calmodulin (CaM)-dependent manner [20]. This role has been recently been confirmed by the work of Sylvain et al. [41]. They demonstrated that targeting AQP4 effectively reduces cerebral edema during the early acute phase in post-stroke mice using a photothrombotic stroke model. They have also shown a link to brain energy metabolism as indicated by the increase of glycogen levels [41]. AQP4 inhibitors may be beneficial in the early course of stroke (cytotoxic phase) but potentially harmful in the vasomotor phase [42]. Currently, there are also no specific AQP4 inhibitors with clinical utility, and no AQP water channel blocker drugs have been approved for use in humans [20]. The increased AQP4 expression and the redistribution/surface localization can be two different concepts. Previous studies have shown an increased in AQP4 membrane localization in primary human astrocytes which was not accompanied by a change in AQP4 protein expression levels. This mislocalization can be a potential therapeutic target [43,44].

### 5.4. Penumbra as an Important Target of Treatment

Striving to preserve the penumbra zone plays an important role in clinical practice. It is an area of brain with relatively preserved function within which the normal functioning of the brain can be restored by reperfusion. The penumbra area is also known as the “inflammatory penumbra” because this area is infiltrated by cytotoxic T-cells. Noteworthy is the discussion of cellular and inflammatory reactions that take place within the penumbra, as this phenomenon is used in practice. When qualifying for stroke treatment using mechanical thrombectomy, perfusion imaging is performed to assess the extent of the penumbra [45]. The process of lymphocyte infiltration is mediated by integrin CD49d and vascular cell adhesion molecule 1 (VCAM-1) [46]. As a method of pharmacological treatment, attempts were made to use a monoclonal antibody blocking the interaction between Cd49d and VCAM-1 (natalizumab), resulting in a decrease in the extent of lymphocyte penetration into the brain and an impact on the clinical course in patients with multiple sclerosis [47]. A very good clinical effect was obtained in a recent Phase 2b study in stroke patients with the safety profiles maintained [48]. Another drug to be assessed for efficacy in the penumbra region was fingolimod, acting via the sequestration of circulating mature lymphocytes in the lymph nodes. The use of this drug inhibits the penetration of lymphocytes into the brain and reduces the ischemic area, but this effect is observed too late [49,50]. The anti-inflammatory effect at the stage of the existence of the penumbra, i.e., in the first 24 h after the onset of a stroke, may be a grasping point in attempts to use lipoxins in the treatment of patients with stroke.

### 5.5. Future Directions of Stroke Treatment

One of the possible (to use large biopharmaceuticals such as therapeutic antibodies) in the in vitro model is transcytosis in a microfluidic device. Such a model involving human brain endothelial cell lines, astrocytes, and pericytes on a two- or three-band microfluidic platform was tested by Wevers et al. [51]. Other authors have developed a microvascular model using the microvascular endothelial cells of the human brain to control the flow of imaging modalities such as transmission electron microscopy (TEM), 3D live fluorescence imaging using traditional spinning disk confocal microscopy, and advanced lattice light sheet microscopy (LLSM) [52]. In the longer term of treatment, stem cell-based therapy has been proposed as an exciting regenerative medicine strategy for brain injury. Cerebral organoids (CO) have been developed as a promising source of transplantation in the event of stroke. Transplanted COs show the potential of multilineage differentiation to mimic in vivo cortical development, support motor cortex region-specific reconstruction, form neurotransmitter-related neurons, and achieve synaptic connection with host brain via in situ differentiation and cell replacement in stroke. Cells from transplanted COs show extensive migration into different brain regions along the corpus callosum [53].

## 6. Arachidonic Acid Derivatives

Arachidonic acid (AA) is synthesized from linoleic acid (LA), which is one of the essential fatty acids. The eicosanoids that can affect intracellular and extracellular signaling pathways, affect gene expression, physiological and metabolic responses in cells and tissues, are derivatives formed through the activity of lipoxygenases [54]. Their role is particularly evident in pro-inflammatory response. There are few studies regarding the effects of increased dietary AA intake on inflammation, especially in humans. However, the few studies conducted show that increasing AA consumption causes an increased amount of AA in cell membranes and increased production of the AA-derived lipid mediators [54]. Apart from AA derivatives formed by lipoxygenase, other mediators are metabolized with the involvement of cytochrome P450 cyclooxygenase (COX) such as prostaglandins, thromboxanes, and leukotrienes [55]. Prostaglandins (PGs) exhibit a number of pro-inflammatory effects, including fever, vascular permeability, and the pro-inflammatory cytokine Il-6. Leukotrienes, on the other hand, take part in inflammatory processes by inducing contraction of arterioles and bronchi and increasing vascular permeability, mucus secretion, and hypersensitivity [54].

Lipoxins are resolving mediators derived from AA, and they promote the resolution of inflammation, microbial clearance, and tissue regeneration [11,12]. Research shows that these are one of the most important derivatives of fatty acids that improve the patient’s condition after ischemic stroke [55]. They act by inhibiting the production of peroxides and inhibiting the transmigration of polymorphonuclear leukocytes [56]. The role of lipoxins in ischemic stroke is related to the reduction of the amount of reactive oxygen species, the production of pro-inflammatory cytokines and chemokines, and blocking adhesion and transmigration through the endothelium [57]. They are also involved in the induction of apoptosis, increase the level of anti-inflammatory interleukins, and promote the resolution of inflammation [57]. It seems that inflammation control is a new strategy of reducing damage caused by ischemic stroke [58].

## 7. Synthesis of Lipoxin A4 (LXA4)

**Figure 1:**
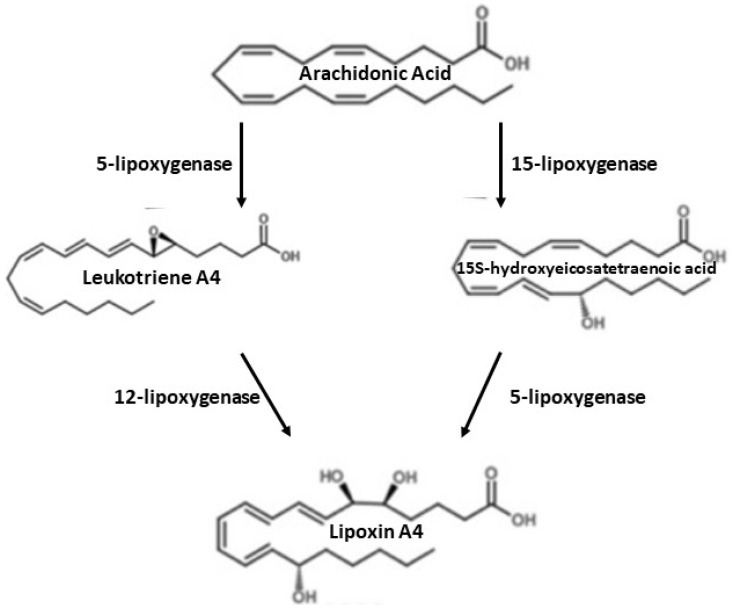
Synthesis of lipoxin A4.

There are two main routes of lipoxin synthesis in human cells derived from peripheral platelets or polymorphonuclear leukocytes (PMN) [58]. In the case of lipoxins synthesis from peripheral blood platelets, 15-lipoxygenase introduces oxygen to AA, which leads to the production of 15-hydroxyeicosatetraenoic acid (15 (S)-HETE), which is converted to lipoxins by 5-lipoxygenase (5-LOX) [58]. In the lipoxin synthesis by polymorphonuclear leukocytes, 5-lipoxygenase converts AA into leukotriene A4, which is then released and converted by platelets to lipoxin A4 by lipoxygenase 12 [58,59,60]. This process is shown in the diagram below (Figure 1). Lipoxin synthesis is also possible with the participation of aspirin (acetylsalicylic acid). Aspirin, such as statins, is the main drug used in the secondary prevention of stroke [59]. Aspirin is a persistent inhibitor of COX-1, it can also modify COX-2 products such as 15-HETE, HEPE, and 17(h)DHA, which are precursors of proliferative mediators [57,60]. With the participation of aspirin, aspirin-dependent lipoxins (ATL) are generated [58,61]. Inflammatory stimuli (e.g., TNF-α, LPS) increase COX-2 activity in endothelial or epithelial cells. After the administration of aspirin, COX-2 undergoes acetylation, is still active, but changes its products from prostanoid precursors to the ATL precursor, i.e., 15(R)-HETE. Then, 15(R)-HETE is converted by 5-LOX into LXA4 or ATL [62].

## 8. Lipoxin A4

Eicosanoids are biologically active lipids, and their levels depend on both physiological and pathophysiological conditions [58]. These mediators are rapidly generated at sites of inflammation and act through specific receptors that initiate signal transduction that leads to coordinated cellular responses to specific stimuli [58,63]. Growing evidence points to the key role of many eicosanoids produced by lipoxygenases, epoxygenases and non-enzymatic pathways in cardiovascular diseases [58].

Eicosanoids include derivatives of AA, which are released from cellular phospholipids and then, with the participation of lipoxygenase, transformed into lipoxin A4 [58]. LXA4 is an endogenous, anti-inflammatory lipid mediator, which is formed from AA as a result of transcellular oxidation by lipoxygenase [64]. LXA4 binds to the G protein-coupled ALX receptor (FPRL1-FPR2) [64]. Moreover, LXA4 also plays a role in estrogen-responsive endometrial cells [65]. LXA4 binds directly to estrogen receptors (ER), where it can influence changes in transcriptional activity, alkaline phosphatase activity, or the expression of genes regulated by estrogen. In addition, LXA4 modulates the proliferation of endometrial epithelial cells through the ER. These processes are mediated by receptors, including CysLT and FPR2/ALX [65]. The ALX receptor is found in many tissues and cells, including neutrophils, monocytes, macrophages, endothelial cells, microglia, and neural stem cells [66,67,68]. Depending on the type of ALX cell, LXA4 can have different effects. The activation of ALX in neutrophils reduces the amount of free oxygen radicals and the production of pro-inflammatory cytokines and chemokines, blocks the adhesion and transmigration of polymorphonuclear lymphocytes through the endothelium, and induces apoptosis [69,70]. Lipoxin A4 bounded with ALX on macrophages derived from monocytes induces the phagocytosis of apoptotic leukocytes, which is another step promoting inflammation resolution [71]. To facilitate the resolution of inflammation, it is desirable that the removal of apoptotic cells does not result in the release of pro-inflammatory mediators from phagocytes [71]. In LXA4-triggered phagocytosis, there is neither release of monocyte chemotactic protein-1 (MCP-1), which plays a role in the development of cardiovascular disease, nor pro-inflammatory Il-8 or other cytokines and chemokines [71,72]. Research results also indicate that lipoxins can inhibit migration and increase the apoptosis of neutrophils [73,74]. Moreover, some in vivo studies have also shown that lipoxins can inhibit the production of reactive oxygen species [75,76].

## 9. Receptors for Lipoxins

The lipid mediators such as lipoxins and resolvins exert their proresolving effects through specific G-protein coupled receptors (GPCR). So far, four GPCR have been identified as the receptors for LXA4 and D- and E-series of resolvins. These GPCRs are ALX/FPR2, DRV1/GPR32, DRV2/GPR18, and ERV1/ChemR23. Recent studies in atherosclerotic mouse revealed a major role for pro-resolving receptors in atherosclerosis [77].

ALX/FPR2 ligates the lipid mediators such as LXA4, aspirin-triggered LX (ATL), resolvins and annexin A1 protein [71]. ALX/FRP2 is also activated by anti-bacterial peptides and amyloidogenic peptides. So, the other classes of ligands such as N-formylated peptides serum amyloid A and cathelicidin LL-37 causes pro-inflammatory signaling through the same receptor [77].

LXA4 has a high affinity for binding to the GPCR, the lipoxin A4 receptor—ALX, which is also referred to as FPRL1/FPR2 (formyl peptide receptor like-1/N-formyl peptide receptor 2) [55]. The activation of ALX reduces the recruitment of neutrophils, increases the production of anti-inflammatory factors, and promotes the removal of inflammatory residues [71,78,79,80]. ALX is expressed in neutrophils, monocytes, and macrophages, as well as in resident brain astrocytes, microglia, and neural stem cells, suggesting that these cells may be a target for the anti-inflammatory action of LXA4 in the brain [76]. ALX/FPR2 has been identified in macrophages in human atherosclerotic lesions [77]. Research shows that mice without homolog of ALX/FPR2 show exacerbated inflammatory response after cerebral ischemia and reperfusion. ALX/FPR2 is also upregulated during myocardial ischemia. Its ligands limit myocardial necrosis and inflammation after coronary artery ligation [77].

ALX/FPR2 also has the ability to interact in vitro with small peptides and proteins that produce signaling responses other than endogenous LXA4 or ATL ligands [28,80]. Studies regarding the ligand binding have shown that LXA4 also interacts with the cysteinyl leukotriene-1 (CysLT1) receptor by acting as a partial agonist [80]. The CysLT1 receptor in the lungs causes airway smooth muscles contraction and the secretion of mucus. Moreover, the low expression of this gene in the heart (in the pericardium) has been demonstrated [81]. LXA4 mediates its action in various tissues and cell types, not only in leukocytes [28,81]. Thus, LXA4 interacts with at least two classes of G protein-coupled receptors—one specific only for LXA4, present on leukocytes (ALX/FPR2), and the other common to LTD4, thus signaling the displacement of PMNs [82].

## 10. LXA4 and Ischemic Stroke

Research shows that the administration of LXA4 methyl ester (LXA4ME) reduces the amount of pro-inflammatory cytokines, tumor necrosis factor-α (TNF-α), interleukin-1β (Il-1β), and increases anti-inflammatory cytokines: IL-10 and transforming growth factor-β1 (TGF-β1) after cerebral ischemia [5,82,83]. Treatment with LXA4ME after cerebral ischemia significantly reduced the neurological deficit and the size of the stroke. Moreover, neuronal apoptosis caused by ischemic injury also is decreased [81].

However, the effects of LXA4 in early brain injury following SAH have not been studied in humans. Guo et al. investigated the role of LXA4 in the rat SAH model. Studies have shown that high doses of exogenous LXA4 reduced brain edema, preserved the integrity of the BBB, and improved neurological outcomes, as well as spatial learning and memory capacity. Silencing the FPR2 receptor by siRNA abolished the beneficial effects of LXA4 [5]. LXA4 had a beneficial effect by inhibiting neutrophil infiltration, inhibiting the expression of phosphorylated p38 protein and the pro-inflammatory cytokines IL-1β and IL-6 in both the cortex and the hippocampus [5]. This is enabled by down-regulating the activity of the mitogen-activated p38 kinase mediated by the LXA4 receptor, FPR2. This study proves that LXA4 provides neuroprotection in early brain damage by inhibiting inflammation in the FPR2/p38 signaling pathway, which has also been demonstrated in other studies [5,84,85,86]. Multiple studies have also shown that the direct injection of LXA4 into the brain immediately after ischemia has reduced the size of the stroke and lowered neuroinflammatory factors in rats [85,86,87,88]. Post-ischemic neutrophil infiltration is a component of the neuroinflammatory response. When neutrophils enter an ischemic brain, they damage tissues by releasing oxygen free radicals, proteases, and phospholipases because the brain contains a large amount of unsaturated fatty acids that are susceptible to lipid peroxidation caused by free radicals [89].

Wu et al. investigated the effect of LXA4ME on BBB permeability, metalloproteinase-9 (MMP-9) activity, and neuronal damage in a rat model of middle cerebral artery occlusion (MCAO) [86]. The results indicate that LXA4ME treatment is effective in improving BBB integrity and reducing stroke volume following focal ischemia. In addition, the activity and expression of MMP-9 were reduced after LXA4ME was administered to rats that were subjected to a 2-h MCAO followed by a 24-h reperfusion [87]. The increase in metallopeptidase inhibitor 1 (TIMP-1) after LXA4ME treatment could contribute to the observed reduction in MMP-9 expression. Stroke was detected by triphenyltetrazolium chloride (TTC) staining. BBB dysfunction was determined by examining cerebral edema and Evans blue extravasation [87]. Moreover, for LXA4ME, studies have shown a strong anti-inflammatory effect [87]. Similar to some anti-inflammatory drugs, LXA4ME also alters the expression and function of MMP-9 and TIMP-1. LXA4ME, similar to aspirin, inhibits the activity of MMP-9 by inducing TIMP-1 expression in mice [90]. Thus, the inhibition of MMP-9 by LXA4ME may be due to the enhancement of TIMP-1 expression [86,87]. Therefore, research results show that LXA4ME reduces brain damage after stroke by improving BBB function, reducing MMP-9 expression, and increasing TIMP-1 expression [87].

## 11. BML-111—Lipoxin A4 Analogue

LXA4 is quickly inactivated in the body, so its strong analogues have been synthesized, such as BML-111 [89]. BML-111 is 7-trihydroxyheptanoic acid methyl ester and is a commercially available LXA4 analog and ALX receptor antagonist. BML-111 is a factor that reduces inflammation, neutrophil infiltration, and inhibits PMN migration [75,82]. BML-111 has been shown to increase the amount of anti-inflammatory agents in some inflammatory diseases, including arthritis, liver, and lung damage [89,90,91,92].

Hawkins et al. assessed the effect of BML-111 in a rat model of focal cerebral ischemia. Forty-eight h after the injection of BML-111, it was checked whether the inflammatory effects of ischemic stroke were reduced [76]. The results of the research showed that after the administration of BML-111 after cerebral ischemia, the protein of the closing zone (ZO-1) was protected against degradation [76]. This suggests that BML-111 reduces inflammatory damage in the cerebral cortex and helps maintain BBB integrity following an ischemic stroke. The results showed that treatment with BML-111 significantly reduces the size of stroke and protects the cerebral cortex, possibly by reducing BBB permeability, reducing MMP-9 and MMP-3, reducing microglia activation, and protecting tight junctions [76]. Studies have not shown that administration of BML-111 reduced the size of the stroke in the striatum cells—the structure of the basal forebrain nucleus [76].

Hawkins et al. in their research hypothesized that intravenous BML-111 treatment after ischemia would provide neuroprotective protection after stroke in rats. The examined rodents were divided into groups receiving 0.3, 1, or 3 mg/kg BML-111 for a period of 24 h, 48 h, or 7 days after ischemic stroke [93].

**Figure 2:**
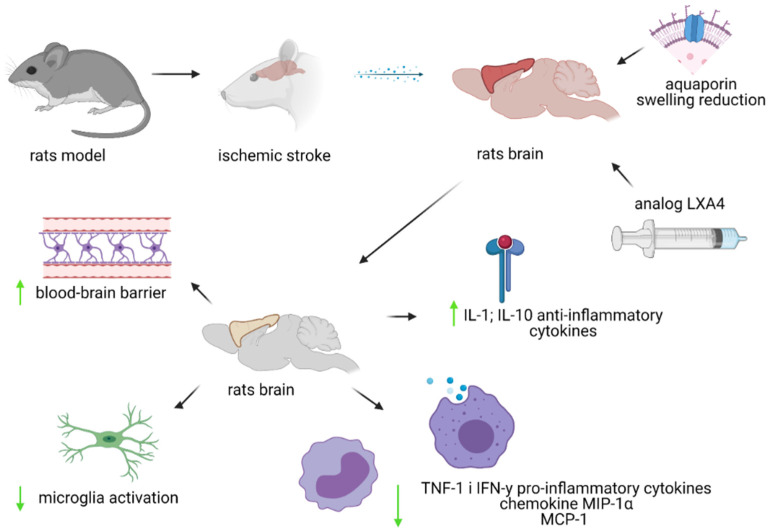
Diagram of the influence of the LXA4 analogue on ischemic stroke in the rat model. IL-1 and IL-10—interleukein 1 and 10; TNF-1—tumor necrosis factor-α; IFN-ɤ—interferon gamma; MIP-1α—macrophage inflammatory protein-1α; MCP-1—monocyte chemotactic protein-1.

No changes in the levels of cytokines and chemokines were observed after the administration of BML-111 24 h after stroke. However, studies have shown that BML-111 administered once a day for 48 h after a stroke lowered the levels of pro-inflammatory cytokines tumor necrosis factor-α (TNF-1) and IFN-y, as well as the chemokines MIP-1α (macrophage inflammatory protein-1α) and MCP-1. Moreover, the administration of BML-111 induced an increase in the level of anti-inflammatory cytokines—Il-1 and Il-10 [92]. In contrast, daily injections of BML-111 for 7 days after ischemic stroke had a positive effect on the size of the stroke and the cellular and molecular profile, but these effects are not sufficient to provide protection 4 weeks after stroke [93]. In studies conducted in a rat MCAO model, one week of injection did not significantly reduce the extent of stroke damage in the cerebral cortex or the total area of the stroke at 4 weeks after stroke [93]. There is a need for further research in this area to see if a longer administration of BML-111 will have a long-term effect on reducing inflammation caused by ischemic stroke [93]. The research results also suggest that the therapeutic effect of BML-111 ensures maximum neuroprotection, and the side effects are minimal [88]. However, when administered at a dose of 3 mg/kg, BML-111 inhibits repair and regeneration processes. The beneficial effects 7 days after stroke in the tested animals (rats) were observed at doses of 0.3–1 mg BML-111/kg [93]. The present state of knowledge is shown below in Figure 2.

## 12. Conclusions

Uncontrolled inflammation plays an important role as a pathogenetic factor of cardiovascular disease such as ischemic stroke. Inflammation control is a new strategy for reducing damage from ischemic stroke. Anti-inflammatory mediators play an important role in controlling inflammation. Lipoxins are pro-resolving mediators that promote the resolution of inflammation, microbial clearance, and recovery. Research confirms the beneficial effect of lipoxins on reducing inflammation through the synthesis of anti-inflammatory mediators and inhibition of the synthesis of pro-inflammatory mediators, improving the prognosis of patients after stroke. These studies were mainly conducted in rodents. The process of suppressing inflammation is achieved by reducing neutrophil infiltration, protecting the cerebral cortex, reducing the permeability of the BBB, and protecting tight junctions, as well as inhibiting the synthesis of pro-inflammatory cytokines and enhancing the synthesis of anti-inflammatory cytokines. However, further clinical studies are needed to establish the benefit in humans and the efficacy, pharmacokinetics, and safe dose to support treatment after stroke. It also should be taken into consideration that LXA4 may directly or indirectly interact with drugs that are commonly used in stroke, such as aspirin or statins.

## Data Availability

The literature was searched with the use of PubMed and Embase databases using a combination of the terms ‘stroke and lipoxin’ and ‘stroke and atherosclerosis’. All articles published up to 31 January 2021 were included in the review. Studies that were not in English, articles duplicated in both databases, letters to the editor, and conference abstracts were excluded from the analysis. A total of 118 articles were analyzed on the basis of which the review was written.
